# Educational intervention to reduce disease related to sub-optimal basic hygiene in Rwanda: initial evaluation and feasibility study

**DOI:** 10.1186/s40814-017-0155-6

**Published:** 2017-06-20

**Authors:** Margaret A. Stone, Hormisdas Ndagijimana

**Affiliations:** 1 Thare Machi Education, P. O. Box 4040, Leamington Spa, CV32 5YJ UK; 2 One Light Rwanda, P.O Box 202, Musanze/Ruhengeri, Rwanda

**Keywords:** Hygiene, Health education, Rwanda

## Abstract

**Background:**

Despite a global reduction in morbidity related to sub-optimal water, sanitation and hygiene, the incidence of such diseases remains a significant problem in sub-Saharan Africa. This study aimed to initially assess the potential effectiveness (primarily in terms of impact on morbidity) of a simple educational intervention delivered in Rwanda. Additionally, we sought to explore feasibility relating to the practicality of evaluating and implementing this type of intervention in a low- and middle-income country.

**Methods:**

Two districts in Northern Province were purposively selected; one was randomly allocated to receive the intervention, with the other acting as control. The intervention was based on an interactive DVD about basic hygiene. Baseline and follow-up data for incident cases of relevant morbidities were collected from health centre records. Changes were compared between the two districts using descriptive statistics and chi-squared tests. Qualitative data were obtained through observations, discussions and feedback and were analysed thematically.

**Results:**

Cases of infection with intestinal worms and parasites were frequently recorded in both districts. For these morbidities, there was a 39% decrease in cases between baseline and follow-up in the intervention district (4995 reduced to 3069), compared to 13% (5002 reduced to 4356) in the control district (*p* < 0.001). Numbers of cases recorded as diarrhoea or dysentery, and changes between baseline and follow-up, were much lower (intervention district 1274 cases reduced to 1171; control district 1949 reduced to 1944). Qualitative data indicated a high level of acceptability for the intervention and also feasibility relating to the practicality of evaluation and implementation, whilst also highlighting problems encountered and possible solutions, in particular, the potential advantages of training local personnel to deliver this type of intervention.

**Conclusions:**

This small-scale study has a number of acknowledged limitations which would need to be addressed in a larger study in order to confidently confirm the effectiveness of the intervention. It nevertheless provides evidence suggesting that the educational intervention is promising in terms of a potential impact on health and feasible to deliver and evaluate. These findings indicate that further evaluation and possibly early implementation are warranted.

**Trial registration:**

Research Registry, researchregistry2180

## Background

In recent years, there has been a shift in the global burden of disease, with a reduction in morbidity related to sub-optimal water sources, sanitation and hygiene and an increase in chronic diseases linked to risk factors such as poor dietary practices and physical inactivity [1]. There are nevertheless large between-country variations, with the World Health Organization (WHO) reporting particularly slow progress relating to water, sanitation and hygiene in sub-Saharan Africa [2]. Young children, in particular, are at high risk for morbidity and mortality linked to diarrhoea [3]. Estimates for Rwanda suggest that, despite a decrease in deaths from diarrhoeal diseases since 2000, such diseases were the fourth most common cause of death, after respiratory disease, HIV/AIDS and stroke [4].

Mortality associated with sub-optimal water supply, sanitation and hygiene is not limited to diarrhoeal diseases. The WHO has suggested that, in 2004, 881,000 non-diarrhoeal deaths were attributable globally to poor quality water, sanitation and hygiene, including 15,000 deaths from the parasitic worm infection schistosomiasis and 12,000 from intestinal nematode infections [5]. In a systematic review and meta-analysis, Ziegelbauer and colleagues confirmed the link between infection with intestinal worms and sub-optimal sanitation access and usage [6].

Low rates of handwashing on key occasions such as following defecation have been observed in low- and middle-income countries (LMICs) such as Ghana [7]. A study investigating bacterial hand contamination amongst Tanzanian mothers highlighted the difficulty of avoiding contamination where good water and sanitation provision is lacking [8]. A 27% reduction in the risk of diarrhoea was identified in Eritrean households where a toilet facility was available [9]. Improving sanitation and water supply in LMICs is clearly important for reducing rates of diarrhoeal disease [10]. There is also, however, a role for education promoting optimal use of the facilities available, although evidence regarding the impact of such education is limited and inconsistent. Positive results were obtained, for example, in a school-based study in rural China, in which an educational intervention increased knowledge and reduced rates of infection with soil-transmitted helminths [11] and in a study in Zaire in which an educational intervention reduced the incidence of diarrhoea in young children [12]. Negative results have also, however, been reported. Following a knowledge and awareness campaign in India, for example, there was no observed increase in handwashing with soap [13]. Even where a statistically significant impact has been shown, this may be very modest, as demonstrated in a school-based study in Kenya promoting safe water, in which there was an increase from 6 to 14% of parents treating their water [14]. Increased levels of knowledge do not necessarily lead to behaviour change, as recognised in a study which used emotional drivers such as nurture and disgust [15]. Even in countries where there are high levels of education, behaviour relating to hygiene is far from optimal; in a UK study, observations suggested that child-carers washed their hands with soap on only 42% of occasions after changing a baby’s nappy [16].

Despite these limitations regarding the evidence that educational interventions are effective, it appears that additional investigation is warranted, especially in relation to LMICs. A recently updated systematic review and meta-analysis of trials assessing the effectiveness of handwashing promotion interventions led to the conclusion that such promotion reduces episodes of diarrhoea. This included a reduction in incidence of around one quarter (incidence rate ratio 0.72, 95% confidence interval 0.62–0.83) identified from pooled results from community-based trials in LMICs [17]. The authors identified 22 trials meeting their inclusion criteria, but these were of variable quality and a high proportion was based in high-income countries. There was only one study from sub-Saharan Africa published over 20 years ago [12]. The current study was developed to assess the potential effectiveness of a simple educational intervention on the topic of basic hygiene (including water and sanitation), delivered in rural Rwanda. We also sought to explore the feasibility of delivering and evaluating such an intervention in a LMIC.

## Methods

### Approvals, timing and study design

Approval was obtained from the Rwanda National Ethics Committee in January 2015. Data collection and delivery of the intervention were conducted between February and September 2015. This was a controlled pilot and feasibility study. The decision to make comparisons at the district rather than the village or community level was based on minimising the risk of between-site contamination. The sample size of two districts was based on practicality for a pilot-level study rather than using a power calculation. Randomisation was used to determine which of the two districts would receive the intervention, but blinding was not possible due to the nature of the intervention. A small-scale qualitative component was included; this was designed to explore feasibility and contribute to the evaluation of impact.

### Setting

The two districts selected were Gicumbi and Rulindo, Northern Province, Rwanda. To minimise any impact of confounding factors, these districts were purposively (rather than randomly) selected for comparability. This included similarity in terms of rural setting, population density, seasonal climate, occupational activities and income levels of people living in the villages. Comparability was determined using reported data [18] supplemented by local knowledge. In addition, people living in these two districts had not been previously exposed to education involving the intervention DVDs. Difficulty of travel for people living in villages in Rwanda suggested a low likelihood of contamination between the two districts through sharing of the educational messages in the intervention. People living in villages in these districts generally have to walk considerable distances to collect piped water, which then requires purification such as boiling to be safe for drinking. At the time of setting up the study, 19 health centres in each of the two districts were identified as venues for showing the DVDs. In total, the populations served by these health centres were 346,276 and 288,452 for Gicumbi and Rulindo respectively.

### Aims and outcome measures

The overall aims of the study were to explore the potential effectiveness of the intervention in terms of any impact on related morbidity and mortality and to assess the feasibility of evaluating and implementing this type of educational intervention in a LMIC such as Rwanda. The primary quantitative outcome measure was change in morbidity linked to sub-optimal hygiene, based on the number of recorded cases of diseases such as diarrhoea and worm infestation. We also sought to explore whether we could identify any changes in relevant morbidity. Assessment of feasibility was exploratory, including consideration of levels of difficulty associated with delivering the intervention.

### Quantitative data collection

A small team of fieldworkers was recruited and trained in Rwanda for involvement in data collection and intervention delivery. Review of the information reported in *Health Center and Dispensary Monthly HMIS Report* forms was used to finalise a standardised data collection form. This form is completed by health centres and returned manually and/or electronically to district offices. In order to focus on incident cases, we selected items of data included in the form which were categorised as ‘new’. We included categories considered to be most relevant to basic hygiene including water and sanitation (Table 1). Aggregated data were extracted at district offices, with no links to individual cases.

**Table 1:** Summary of data collected for baseline and follow-up periods from health centre records in intervention and control districts

Items of data (numbers of new cases) collected and used for measuring changes in morbidity and mortality: • Dysentery/diarrhoea in adults and in children aged 5–19 years (any cases recorded as diarrhoea with dehydration, diarrhoea no dehydration or diarrhoea bloody (dysentery)) • Food poisoning in adults and in children aged 5–19 years (any cases recorded as food poisoning) • Worms/parasites in adults and in children aged 5–19 years (any cases recorded as schistosomiasis, *Ascaris lumbricoides*, *Trichuris trichiura*, hookworm, *Entamoeba*, *Giardia* or *Taenia*) • Dysentery/diarrhoea in infants aged 0–4 years (cases recorded as diarrhoea with severe dehydration, diarrhoea with evident signs of dehydration, diarrhoea no dehydration, diarrhoea bloody (dysentery), persistent diarrhoea or severe persistent diarrhoea) • Any deaths recorded for adults, children or infants, with diarrhoea or dysentery given as the cause • Any deaths recorded for adults, children or infants, with food poisoning recorded as the cause	

Baseline data were collected for a 3-month period (November 2014 to January 2015) before the start of the intervention. The same data were collected for a follow-up period comprising the final 3 months during which the intervention was implemented (June to August 2015). Random samples of baseline and follow-up data for both districts were checked for accuracy, and further checks and corrections were made where indicated.

### Intervention

At the end of the baseline data collection period, one district (Gicumbi) was randomly allocated (using Microsoft Excel RAND function) to receive the intervention, with the other (Rulindo) designated as the control. The intervention was delivered over a period of just over 6 months, beginning towards the end of February 2015 up to the end of August 2015. The intervention comprised education sessions using an interactive DVD developed by Thare Machi Education (TME), a small not-for-profit organisation based in the UK (registered charity no. 1080131) [19]. TME’s stated aims include providing access to basic education to people in developing countries, using simple interactive DVDs in local languages, on topics mainly related to health and wellbeing. The DVDs require no reading or writing ability and are designed to be easily understood by people with limited health literacy and educational skills. For our study, the Kinyarwanda language version of the TME Basic Hygiene DVD was used (Table 2).

**Table 2:** Thare Machi Education Basic Hygiene DVD

General format of TME DVDs: The interactive DVDs produced by TME include a combination of information and questions, illustrated using relevant still or moving images. Questions based on the information given are used throughout the DVDs to reinforce messages; each question has a choice of two answers, one of which is selected by the person or group viewing the DVD. Where an incorrect answer is given, the message and question are repeated, and there is a ‘quiz’ at the end of the DVD to further reinforce the messages. Summary of topics covered in the Basic Hygiene DVD: • Germs and how they may be spread • Cleanliness, including handwashing and importance of soap • Preventing infection from human and animal faeces • Importance of keeping babies and small children clean • Cleanliness when people are unwell • Safe drinking water, including purification • Food safety to prevent infection • Cleanliness within the home, including keeping bedding clean • Preventing the spread of germs through coughs and sneezes Sample text from DVD script: Message: * If you are changing your baby’s nappy, make sure you wash your hands afterwards. If your baby or child does a poo near the house, clean it up immediately. Always wash your hands afterwards* Question: *What should you do after changing a baby’s nappy? Wash your hands, or start cooking?* If correct answer selected: *Well done! Always wash your hands after changing a baby’s nappy*. If incorrect answer selected: * That was the wrong answer. Let’s go back and listen again.*	

Education sessions were delivered pragmatically, using a projector or a laptop computer with battery power, depending on the venue and electricity supply. Fieldworkers kept a record of the number of people who attended sessions, using close estimates for large numbers. After the sessions, health centres were provided with one or more copies of the DVD, which they could continue to use if they wished, but information regarding continued use was not formally collected.

### Feasibility testing

At the end of the study, qualitative feedback was collected from a small purposive sample of people who had contributed to the conduct of the study and from people involved in healthcare provision in Gicumbi (Table 3). This stakeholder feedback was collected verbally, with detailed note-taking, by the principal investigator from the UK, with interpretation provided by the principal investigator in Rwanda where required. Information collected in this way was supplemented by observations noted and discussions between the principal investigators during the conduct of the study, including problems encountered.

**Table 3:** Stakeholder feedback: sources of data

*Feedback was collected as follows:* • A small focus group discussion was conducted with three fieldworkers recruited and trained to deliver the intervention and collect data. This discussion also included some input from the principal investigator in Rwanda. • Feedback was obtained through informal interviews with three people involved in healthcare provision in Gicumbi. These people had management, co-ordination and/or direct healthcare roles. • At a training session for community health workers (lay men and women recruited from local villages), some feedback was obtained from those (*n* = approximately 100) who attended.	

### Analysis

Changes in the number of incident cases recorded as diarrhoea and worm infection were calculated for the two districts, by comparing baseline and follow-up data. Percentages for (positive or negative) changes, as a proportion of the number of cases at baseline, were also calculated. A chi-squared test was used to compare the incidence of recorded cases in the intervention and control districts, for the combined age groups studied, for the two time periods. A one-tailed *p* value was determined as there was no expectation that the intervention would significantly increase the types of morbidity studied. We also determined the difference in difference [20] for percentage changes relating to infection with intestinal worms and parasites. Microsoft Excel was used for the quantitative analysis. Analysis of qualitative data involved initial review of the data collected in order to identify key themes. Data were then classified within these themes for further consideration. We adopted an exploratory approach during the process of analysis, but this process was largely deductive, based on addressing our pre-set aims relating to feasibility and potential effectiveness.

## Results

### Quantitative findings

During the intervention period, the Basic Hygiene DVD was viewed by over 47,000 people at sessions delivered by the research team. Those who attended included both men and women from low-income village communities generally reliant on small-scale agricultural activities. They were often accompanied by young children and babies. Initial review of the data identified very low numbers of cases recorded as food poisoning, so the analysis was restricted to impact on diarrhoea and dysentery and worms and parasites. At baseline, similar numbers of cases of infection with intestinal worms and parasites were identified in our two study districts, in the combined age groups for which data were collected (Gicumbi 4995; Rulindo 5002). Much lower numbers of cases were recorded as diarrhoea and dysentery (Gicumbi 1274; Rulindo 1949) (Table 4). There were no deaths recorded as being the result of diarrhoea and dysentery, in either of the two periods for which data were collected.

**Table 4:** Summary of changes in the numbers of cases of diarrhoea and worms and parasites between baseline (November 2014–January 2015) and follow-up (June–August 2015), at health centres in Gicumbi and Rulindo

	Gicumbi (intervention district)				Rulindo (control district)				
	Baseline	Follow-up	Change	% change	Baseline	Follow-up	Change	% change	*p* value^1^
Diarrhoea in adults^a^	598	459	−139	−23	795	527	−268	−34	
Diarrhoea in children^b^	262	270	+8	+3	445	353	−92	−21	
Diarrhoea in infants^c^	414	442	+28	+7	709	1064	+355	+50	
All diarrhoea	1274	1171	−103	−8	1949	1944	−5	0	0.072
Worms and parasites in adults	3779	1875	−1904	−50	3444	2927	−517	−15	
Worms and parasites in children^b^	1216	1194	−22	−2	1558	1429	−129	−8	
All worms and parasites	4995	3069	−1926	−39	5002	4356	−646	−13	<0.001

^1^Comparison of numbers of cases (all ages studied) between intervention and control districts and between time periods before and during implementation of the intervention

^a^Ages 20 years and over

^b^Ages 5–19 years

^c^Ages 0–4 years

For diarrhoea and dysentery, combined results for adults, children and infants indicated a decrease from 1274 to 1171 cases (8%) in the intervention district (Gicumbi), but a difference of only 5 cases in the control district (Rulindo). Comparison of numbers of cases in the two districts in the two time periods indicated borderline non-significance (*χ* ^2^ = 2.51, *p* = 0.072). For worms and parasites, comparison of rates of change between baseline and follow-up revealed a much greater between-district difference in difference. In Rulindo, there was an overall reduction from 5002 to 4356 cases (13%), assumed to be due to factors unrelated to the intervention, such as seasonal variation between baseline (November to January) and follow-up (June to August) periods. In Gicumbi, the number of cases fell from 4995 to 3069 (39%). It was assumed that a reduction of 26% (39% less 13% due to other factors) is likely to be linked to the impact of the intervention. Comparison of results relating to worms and parasites in the two districts indicated statistical significance (*χ* ^2^ = 127.68, *p* <0.001) (Table 4).

### Qualitative findings

Qualitative data could be grouped into three broad themes. Since data collection involved written notes rather than audio-recording, paraphrasing as opposed to presentation of direct quotations is used below to illustrate key findings within these themes.

#### Acceptability to recipients and providers of the intervention

High levels of engagement were observed in those attending the viewings, and the interactive format of the DVD and the simple messages appeared to be very well received. One fieldworker who attended the focus group described how, after viewing the DVD, some of the men turned to the women and asked if they had been listening carefully. Feedback indicated that people consistently stayed for the entire session and often asked for the fieldworkers to come again. In the focus group, fieldworkers indicated that their own involvement had been a rewarding experience because of the positive reactions of those who viewed the DVD.

#### Effectiveness

People involved in healthcare provision who were interviewed indicated anecdotally that positive changes had been observed in relation to hygiene practice and cases of related diseases. One also described how the DVD had been a useful training aid for community health workers (lay members of village communities), strengthening their capability for teaching people within their communities. This was confirmed by the community health workers who attended the training session at which feedback was invited. A question about whether they had found the DVD useful elicited an enthusiastic positive response, accompanied by broad smiles. They explained that the DVD had given them additional information about key messages that they needed to disseminate within their villages.

#### Feasibility

Carrying out the research was described and observed as feasible, but reported challenges included problems with power supply and difficulties with travel and transport due to poor terrain and weather conditions. Fieldworkers described how visits to health centres had sometimes involved up to 4 h of travel each way. They also reported problems with power cuts or lack of a good electricity supply in some remote settings. Exploration of solutions to these problems during our interviews and discussions highlighted the potential benefits of using local personnel for evaluating and potentially implementing the type of intervention being investigated. It was suggested, for example, that Information, Education and Communication sessions could be used for showing DVDs at health centres or that community health workers could be provided with small battery-operated DVD players for showing DVDs within their villages.

## Discussion

### Impact

Our pilot-level study confirmed high levels of need in terms of reducing the morbidities studied. It also demonstrated the potential effectiveness of the intervention being tested. Our findings regarding high numbers of cases of intestinal worms and parasites in our study districts support previous observations regarding the high global burden of worm infestation, including particularly high rates in sub-Saharan Africa [21]. The DVD used for the intervention includes messages about hygiene including the spread of infection via soil, water and food and preventive measures including handwashing, water treatment and safe disposal of faeces. Such messages are highly relevant to preventing transmission of infection with worms and parasites.

We used a controlled design that would help us to assess the extent to which changes were likely to be linked to the intervention or other factors such as seasonal variation. It is possible that there were unidentified confounding factors which influenced the difference in percentages relating to changes in the incidence of infection with worms and parasites in the two districts. These districts were, however, purposively selected for similarity, and we are not aware of any events or other interventions which are likely to have influenced the numbers of cases during the intervention period. Although we had been unable to obtain formal quantitative information about deaths related to intestinal worms, this issue was discussed as part of our feedback data collection from individuals involved in healthcare provision in Gicumbi. We were informed anecdotally that, in the year prior to the start of the study, there had been a small number of related deaths in young children. This suggests that there is potential for a reduction in mortality if morbidity can be reduced through education.

Comparison of changes relating to the numbers of cases of diarrhoeal disease was non-significant, and inconsistencies made findings difficult to interpret. In Gicumbi, for example, a reduction in cases in adults was not matched by results for children and infants. Their findings may, however, be influenced by the types of cases recorded in these categories by health centres, including possible overlap between diarrhoea and worm infestation. Findings for diarrhoea may also be influenced by lack of statistical power due to lower numbers of recorded cases compared to infection with worms and parasites.

Two previous studies had provided some evidence regarding the impact of the TME DVDs. An evaluation conducted in six countries indicated an increase in knowledge after watching a DVD-based lesson (unpublished report available on request from TME). Research in the UK demonstrated significant changes in understanding and attitudes amongst people of South Asian origin who viewed a DVD about insulin in type 2 diabetes, produced as a collaboration between TME and the University of Leicester [22]. Although these evaluations provided promising evidence, no hard outcomes relating to morbidity were included. Results from the current pilot-level study suggested a clinical impact in relation to cases of infection with worms and parasites, based on health centre data.

There is some evidence that interventions with additional components alongside education may have greater impact. In a quasi-experimental study in Ethiopia, for example, tailored interventions including public-commitment with or without handwashing-station-promotion were more effective in terms of self-reported handwashing than education alone [23]. In comparing educational community-based handwashing interventions in LMICs, Ejemot-Nwadiaro and colleagues identified a greater impact in six trials that provided free soap compared to two that did not. These authors drew attention, however, to the small number of trials on which this finding was based and the difficulty of determining the relative impact of soap provision and handwashing promotion from the limited evidence available [17]. Additional measures within an intervention are likely to have cost implications; in a study in rural Peru aimed at reducing childhood diarrhoea and respiratory disease, which was unable to confirm an impact on health outcomes, an integrated package was provided including water purification bottles, solid fuel stoves and sinks with piped water, in addition to hygiene promotion [24]. In our study, we sought to specifically evaluate, at the pilot level, the potential effectiveness of a low-cost educational approach without additional interventions such as provision of soap.

### Feasibility

Our qualitative findings indicated the practicality of carrying out the type of research investigated and also of potential implementation in a real-world setting. Feedback and observations suggested high levels of acceptability relating to the intervention, but also highlighted the importance of being aware of potential challenges. For practical reasons, the intervention was delivered by a team of fieldworkers who travelled to the health centres in the intervention district, but experiences and feedback suggested that it may be preferable to use local personnel. Training local providers to show the DVDs would provide a practical method of delivery that could lead to sustained impact.

### Limitations

It is acknowledged that this small-scale study has some important limitations which would need to be addressed in a definitive evaluation. Using our data collection methods and working within time and budgetary restrictions, we were unable to collect numbers specifically for deaths from infections with worms and parasites, so it was not possible to quantitatively investigate any impact on mortality from these infections. From the data collected, we were also unable to compare cases of worms and parasites in children aged under 5 years, in whom these infections are particularly common. The health outcome information collected was restricted to recorded cases of relevant diseases in two defined periods, so we are unable to provide evidence related to other indicators such as longer term trends. Potential for statistical testing, to provide a robust assessment of effect size, was restricted by the data collected, including a lack of detailed information regarding the numbers of people in different age ranges served by the health centres. Our between-district comparisons using a chi-squared test were designed to provide an initial indication of effectiveness only but nevertheless suggested a significant impact regarding the incidence of infection with intestinal worms and parasites.

It is further acknowledged that we cannot fully guarantee the accuracy of the data provided in health centre submissions to the district offices, although observations in one health centre prior to the start of the study suggested that information entered on report forms was derived from conscientious recording in paper records of clinic activity. Whilst recognising the small scale and informality of the qualitative aspect of our study, including stakeholder feedback, we consider that this made a useful contribution, particularly in terms of investigating feasibility. This emphasises the usefulness of including this type of component in evaluations both at the pilot and the full study level. It is acknowledged that qualitative data collection by one of the principal investigators could potentially have led to biased feedback, although care was taken not to deliberately influence responses. Furthermore, respondents were willing to share negative reflections relating to problems encountered as well as more positive feedback.

It is clear that any shortcomings within a pilot study need to be taken into account when considering the strength of the evidence provided, and we fully acknowledge that our findings should be viewed bearing in mind the limitations described above. Our results relating to cases of infection with worms and parasites nevertheless raise the question of whether notable findings provided by a pilot study suggest that early implementation of the intervention may be justified in advance of more rigorous evaluation. This is particularly relevant where a high level of need has been confirmed. On this basis, training of community health workers in the use of the TME DVDs has been initiated in Rwanda, with encouragement and facilitation (but not financial support) from the Rwandan Ministry of Health. Over 5000 community health workers received training between June and November 2016, and this programme is currently ongoing (Rachel Butt, Director, TME, personal communication). It is recognised, however, that sustainable financial support, such as government funding, is likely to be needed in order to cover the cost of full-scale implementation of any intervention. Justifiably, such funding is likely to be difficult to secure without confirmation of impact and an economic evaluation that provides strong evidence of cost-effectiveness. Economic evaluation was not within the scope of our pilot study.

## Conclusions

Our study has demonstrated the feasibility of evaluating and implementing a simple educational intervention on the topic of basic hygiene in a LMIC in sub-Saharan Africa. A larger, cluster randomised trial including estimation of sample size and adjustment for clustering would be required in order to confidently confirm the effectiveness of the intervention and to clarify the potential for reducing diarrhoeal disease. More comprehensive data collection would also be required, including health data from additional sources, information about how the educational messages were further disseminated by those who viewed the DVD and assessment of cost-effectiveness. Using local personnel was highlighted as a potentially effective and cost-effective method of delivering the intervention, but further formal evaluation or monitoring would be required to confirm the impact of the educational intervention using this method of delivery. Overall, it is considered that the results of our small-scale, low-budget study suggest that the intervention is promising in terms of a potential impact on health and that further evaluation and possibly early implementation are warranted.

## Abbreviations

LMICs: Low- and middle-income countries
TME: Thare Machi Education
WHO: World Health Organization
